# Recent Progress of Biodegradable Polymer Package Materials: Nanotechnology Improving Both Oxygen and Water Vapor Barrier Performance

**DOI:** 10.3390/nano14040338

**Published:** 2024-02-09

**Authors:** Shuangshuang Yue, Tianwei Zhang, Shuanjin Wang, Dongmei Han, Sheng Huang, Min Xiao, Yuezhong Meng

**Affiliations:** 1 The Key Laboratory of Low-Carbon Chemistry & Energy Conservation of Guangdong Province, State Key Laboratory of Optoelectronic Materials and Technologies, School of Materials Science and Engineering, Sun Yat-sen University, Guangzhou 510275, Chinazhangtw7@mail2.sysu.edu.cn (T.Z.);; 2 School of Chemical Engineering and Technology, Sun Yat-sen University, Guangzhou 510275, China; 3 Research Center of Green Catalysts, College of Chemistry, Zhengzhou University, Zhengzhou 450001, China; 4 China Institute of Chemistry, Henan Academy of Sciences, Zhengzhou 450000, China

**Keywords:** surface-nanotechnology, biodegradable polymers, packaging materials, oxygen barrier, water vapor barrier

## Abstract

Biodegradable polymers have become a topic of great scientific and industrial interest due to their environmentally friendly nature. For the benefit of the market economy and environment, biodegradable materials should play a more critical role in packaging materials, which currently account for more than 50% of plastic products. However, various challenges remain for biodegradable polymers for practical packaging applications. Particularly pertaining to the poor oxygen/moisture barrier issues, which greatly limit the application of current biodegradable polymers in food packaging. In this review, various strategies for barrier property improvement are summarized, such as chain architecture and crystallinity tailoring, melt blending, multi-layer co-extrusion, surface coating, and nanotechnology. These strategies have also been considered effective ways for overcoming the poor oxygen or water vapor barrier properties of representative biodegradable polymers in mainstream research.

## 1. Introduction

In the logistics and transportation process, it is important to properly package the cargo for protection and security purposes. Packaging is the combination of science, technology and art to enclose a commodity for safe transportation and distribution of the commodity to the users at a minimum price [1]. To extend the shelf life and limit wastage of products, packaging materials should have high standards of gas barrier performance: the humidity should be kept within a certain range to avoid dryness of the product, and oxygen concentration must be kept ultra-low to prevent oxidation and degradation of the product. Numerous businesses place a high value on research into the adsorption, transport, and desorption characteristics of oxygen and water vapor in packing materials. These include a wide range of uses, from moisture-proof materials and anti-corrosion barrier films to packaging materials for consumer goods (such as food, medicines, and microelectronics).

The most often used materials in the packaging sector are cans, glass bottles, paper bags, and plastics, with plastics accounting for more than half of the market due to their affordability and superior durability [2]. Because plastic is simpler to make, more inventive, and more cost-effective than metal and glass, it has recently taken the role of these materials. In recent years, the use of glass and metal has been replaced by plastic because it is easier to manufacture, resourceful, and more economical [3]. However, because plastics are naturally permeable to many gases, research has focused on creating novel polymer materials with enhanced barrier properties. The consumer’s need for more convenience, environmental awareness, transparency, lighter and thinner packaging, and fresher food and beverage goods are also contributing to the increase [4].

To improve the barrier qualities of polymer films, a variety of intriguing new technologies have recently developed [5]. The improvements fall into seven categories: Vacuum-deposited inorganic coatings that are thin and transparent; organic coatings; organic/inorganic hybrid coatings; blends of barrier polymers and common polymers; nanocomposite materials; multi-layer composites; and multi-layer co-extrusion materials. A number of technologies and materials have found extensive use in the packaging of food, electrical devices, medicines, everyday needs, and cosmetics.

However, the majority of barrier packaging materials used in commerce today are made of non-degradable polymers such as polyprolylene (PP), polyethylene (PE), polyamide (PA), poly(vinylidene chloride) (PVC), polyethylene terephthalate (PET), ethylene vinyl alcohol copolymer (EVOH), and others. Despite their great availability, strong mechanical qualities, and outstanding gas barrier properties, they cannot meet the demand of an increasingly environmentally conscious society, which mandates the adoption of more ecologically friendly techniques and materials.

Biodegradable plastics, such as cellulose, thermoplastic starch, poly(lactic acid) (PLA), polyhydroxyalkanoates (PHAs), polycaprolactone (PCL), and poly(butylene adipate-co-terephthalate) (PBAT), etc. [5] have significant advantages in the aspects of the environment but have not been widely used in the field of barrier packaging, either because their uneasy to process or because their oxygen or water vapor barrier properties are still insufficient to serve as high barrier packaging materials.

The aim of this paper is to introduce the fundamental principle of barrier materials, summarize the basic ways and methods to improve the barrier properties of package material, and, most importantly, outline different kinds of biodegradable polymers and recent progress in improving their oxygen and water vapor barrier properties.

## 2. Fundamental Principles

### 2.1. Fundamental Principles of Permeation of Gases through Polymer Films

**Figure 1:**
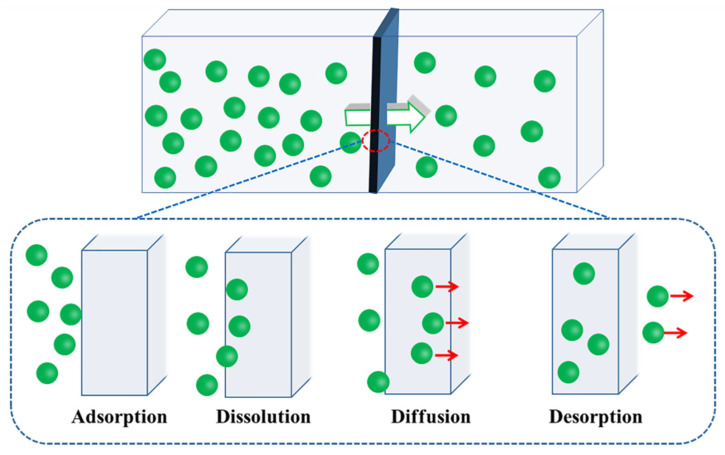
Permeation process of gas molecules in membrane materials.

The fundamental principles describing the permeation of gases through polymer membranes have been discussed in numerous publications [6,7]. Due to the multiplicity of polymer motion units and the creep of polymers, polymers are inherently permeable, so it is difficult to form a complete barrier to gas molecules. Figure 1 shows the permeation process of gas molecules in a polymer membrane material, which is completed in four steps [8]: 1. adsorption of gas molecules on the surface of polymer materials; 2. dissolution; 3. diffusion at a certain concentration gradient; 4. desorption on the other surface of the material. The adsorption and desorption steps of gas molecules on the material surface are relatively fast. Hence, the penetration rate of gas molecules mainly depends on the dissolution and diffusion rate of gas molecules in polymer materials [9].

### 2.2. Representing Method and Testing Method of Barrier Properties of Polymer Materials

#### 2.2.1. Representing Method of Barrier Properties

The transmission rate and permeability of a polymer material can be used to describe its barrier quality to oxygen and water vapor (Table 1). The transmission rate, which varies with the material types and film thickness, is the weight or amount of a permeant (such as water vapor or oxygen) traveling through a film per unit surface area and time under equilibrium testing circumstances. Permeability is the transmission rate multiplied by the thickness, which is considered to be the characteristic constant of a material.

#### 2.2.2. Different Test Methods for Oxygen and Water Vapor Permeability

Oxygen and water vapor barrier properties are the main parameters concerned in packaging applications. According to the test principle, the oxygen permeability test methods are mainly divided into equal pressure methods and differential pressure methods. In the equal pressure method (GBT19789-2021) “Packaging material-test method for oxygen gas permeability characteristics of plastic film sheet-Coulometric sensor”), the test specimen divides the test chamber into upper and lower sides. A nitrogen-filled atmosphere is present on one side, while an oxygen-filled atmosphere is present on the other. Although the pressure is the same on both sides, the partial pressure of oxygen varies. Oxygen passes through the membrane and is directed into the sensor through nitrogen carrier gas as a function of the oxygen concentration differential. When the oxygen level in the nitrogen side environment remains constant, the test is finished. According to the oxygen amount that is precisely detected by the sensor in nitrogen carrier gas, the oxygen transmission rate (OTR) of the film may be determined [10]. Oxygen permeability (OP) is calculated using Equation (1)

OP = OTR × d (1)

where d is the film thickness (mm).

The differential pressure testing method is referred to the national standard GBT1038.1-2022, “Plastic-film and sheet-determination of gas transport-differential pressure method”. The gas percolation cell is separated into two compartments by the test sample, and the air in both chambers is vacuumed to ensure that the static vacuum pressure change in the downstream compartment is smaller than that due to gas diffusion. Then, the upstream compartment was filled with oxygen at a pressure of about 0.1 MPa. The oxygen will penetrate from the high-pressure side to the low-pressure side under a differential pressure gradient, and the oxygen permeability (OP) can be obtained by calculating the internal pressure of the low-pressure side [11].

The water vapor barrier performance test mainly includes the weighing method and the sensor method. The national standard GB/T 1037-1988, “Water permeability test method for plastic film and sheet—cup method”, defines the weighing method. Its principle is under a room of controlled temperature and humidity. Jars are filled up with a certain quantity of water and closed by the sample of interest, which serves as a lid. A metal screw-top rim and silicone gasket seal the sample against the jar. To prevent any early alterations brought on by the humidity conditions, samples are treated for at least one day. The water vapor transfer rate (WVTR) was calculated using the weight change over at least 24 h. The weighing method has the advantages of simple operation and low equipment price, and the unit of WVTR is g/m^2^·24 h [12]. The following Equation (2) is used to calculate the water vapor permeability (WVP) of the films:

WVP = (WVTR × d)/Δp (2)

where Δp is the partial water vapor pressure difference (MPa) across the two sides of the film and d is the film thickness (mm). The unit of WVP is g·cm/(m^2^·s·Pa).

The corresponding national standard for sensor method is GB/T 21529-2008 “Water vapor transmittance determination method for plastic film and sheet-infrared sensor method”. According to different sensors, the sensor method can be divided into infrared sensor method, electrolytic sensor method, humidity sensor method, etc. The cell halves are separated by the specimen. The pressure on either side of the sample is equal, with humidified nitrogen gas on one side and dry nitrogen gas on the other side. Water vapor molecules percolate from the humidified gas side to the dry gas side, and the nitrogen gas carries the water molecules to the sensor, generating an electrical signal to output the date. The new sensor method equipment has the advantages of high precision and good repeatability, and the unit of WVTR is g/(m^2^·24 h). The following Equation (3) is used to calculate the WVP (g·mm/(m^2^·day)) of the films:

WVP = WVTR × d (3)

where d was the film thickness (mm).

### 2.3. Oxygen and Water Vapor Barrier Property of Some Commercial Polymer Package Materials

The oxygen permeability (OP) and water vapor permeability (WVP) are the most important properties of the barrier materials, which are determined mostly by the composition and structure of the polymers. Table 2 summarizes the OP and WVP of some commonly used polymer package materials.

The gas permeability of the polymer films depends on the composition and structure of the film and is particularly correlated to the density, hydrophobicity, and crystallinity [24]. Typically, polymers with high hydrophobicity and low density, such as PP and PE, have excellent water vapor barrier properties but poor oxygen barrier properties. Meanwhile, polymers with high density and abundant hydrogen-bonded networks, such as EVOH et al., show superior oxygen barrier properties but poor water vapor barrier properties in highly humid conditions. PVDC has both excellent water vapor and oxygen barrier properties due to its hydrophobicity and ultrahigh crystallinity and density.

### 2.4. Basic Ways and Methods to Improve the Barrier Property

#### 2.4.1. Blending vs. Multi-Layer Composite

##### Polymer Blending

Blend polymer refers to the physical mixture of two or more polymers. The purpose of blending is to combine the advantages and make up the drawbacks of the individual components so as to prepare polymer materials with improved or unique properties. For example, blending high oxygen barrier materials with high water vapor barrier materials to obtain a blending polymer with both good oxygen and vapor barrier properties. Some of the blends that have been well-developed are PA in PP or PET, EVOH in PP, PE or PA, etc. [25,26,27,28,29]. However, blend films prepared by melt blending often encounter problems such as phase separation. So, novel plasticizers and compatibilizers have been investigated to improve the melting processability of such blends, of which maleic anhydride (MA) [25] and ionomers are usually used as compatibilizers for polymer blends [30].

In addition to the compatibility, the morphology of the polymer blend is also crucial to the barrier properties. Layered blending has been shown to be effective in improving the oxygen barrier property of polymer blends. During the process, one phase can be stretched into micro or nanosheets under an intense flow field, which works as a barrier wall to prolong the diffusion path of oxygen gas in the blends.

##### Multi-Layer Composite

Multi-layer melt blending is a convenient way to develop novel polymeric materials, especially suitable for the phase-separate existing blend system [31]. The synergy between the blended two or more polymer components can lead to enhanced properties of the material. Developing and optimizing processing techniques and processes for preparing multilayers is an important research direction [32]. Multi-layer melt composites can be divided into the multi-layer dry composite method and the multi-layer co-extrusion method.

Multi-layer dry composite method: various membrane or sheet materials are used as matrix materials, coating a layer of viscose on the surface of the substrate after infrared heating and drying and then compounding with other different types of sheets by rolling to prepare multi-functional composite barrier materials [33]. For example, a composite was prepared using a multi-layer dry composite method using polyfluoroethylene and polymer aluminized film as a sheet and solvent-based thermosetting polyurethane as an adhesive. It not only has low gas transmittance but also has good physical and mechanical properties [34].

Multi-layer co-extrusion method: The preparation of high-barrier plastic products using multi-layer co-extrusion is the most commonly used method in industrial production [35]. The technology of producing multi-layer structure composite film by multi-layer co-extrusion is realized by using multiple extruders and a multi-channel composite die. Generally speaking, more than two kinds of polymer materials are fused in a die in a molten state. The compatibility and adhesion of the inner barrier layer and the outer layer are very poor, so it is often necessary to add a bonding layer between the inner and outer layers to firm the inner and outer layers. The plastic products prepared using this method have excellent comprehensive barrier properties and mechanical strength. LDPE/bonding layer/EVOH/bonding layer/LDPE is a typical multi-layer co-extrusion material that is widely used in cosmetics packaging. The barrier performance of the composite film can be improved with an increase in the number of composite layers, which is easy to control [36]. Multilayer co-extrusion techniques can be divided into two broad categories. One is the multi-extruder system (five or more), which allows more than five different polymers to be combined and assembled into a multi-layer structure. This technique has been widely adopted by the industrial sector. However, the drawback of this technique is that the number of layers is limited by the number of extruders [37]. Multi-layer structured films can also be prepared by splitting the melt from a conventional or refurbished feed block using layer multipliers, which can produce films with tens to thousands of layers, while the materials can be only two different polymers plus one surface lamination [38].

#### 2.4.2. Polymer/Inorganic Layered Material Nanocomposites

The concept of nanocomposites proposed by Roy and Kormameni in 1984 is that the composite material of at least one dimension in three-dimensional size of the dispersed phase size is less than 100 nm [39]. The blending of natural layered nanomaterials with polymers often gives the nanocomposites improved mechanical and thermal properties. As for barrier properties, an enhancement is obtained by increasing the tortuosity of the diffusion path.

**Figure 2:**
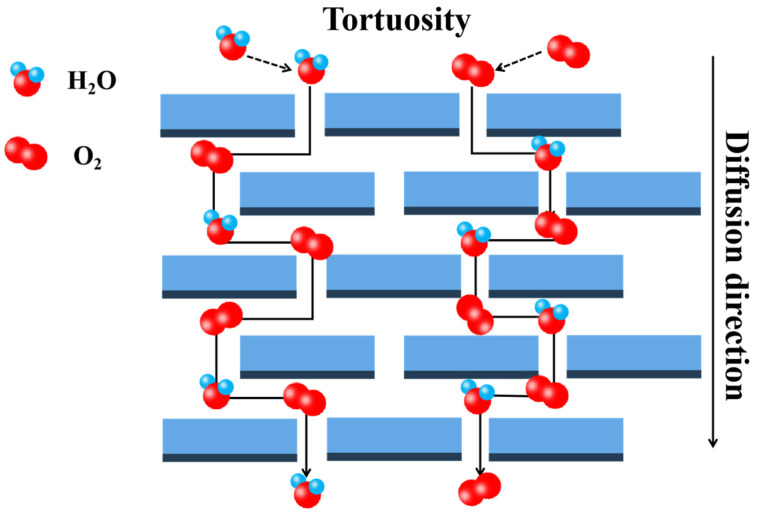
Schematic diagram of the tortuous path model.

A model for the permeability of filled polymer systems was first proposed by Nielsen [40]. This model is based on the premise that the permeability of filler particles increases the diffusion path of the gas throughout the system. Therefore, this model is usually referred to as the “tortuous path” model, as shown in Figure 2. The barrier properties of polymers can be appreciably enhanced by the inclusion of impermeable lamellar fillers (e.g., clay) with a high aspect ratio to prolong the diffusion path of diffusing molecules [41,42].

The commonly used polymer matrix materials are PE, PA, PP, PET, and so on, and the nanomaterials mainly include metals and their oxides, different types of clays, graphene, and so on [43]. The morphology of the nanosheets in the polymer matrix has a significant role in the enhancement of efficiency. There are three morphological types of nanocomposites based on the degree of nanosheet dispersion: aggregated, intercalated, and exfoliated. In the aggregated structure, the lamellar fillers are distributed in the polymer matrix, but the individual fillers are not delaminated. In the intercalated structure, the lamellar fillers are, to some extent, delaminated: the polymer chains can diffuse into the galleries between them. In the exfoliated structure, the lamellar fillers are completely broken up into monolayers and are homogeneously dispersed in the matrix. The exfoliated structure is the most desirable state, as it affords excellent barrier as well as mechanical and thermal properties at very low clay content [44].

**Figure 3:**
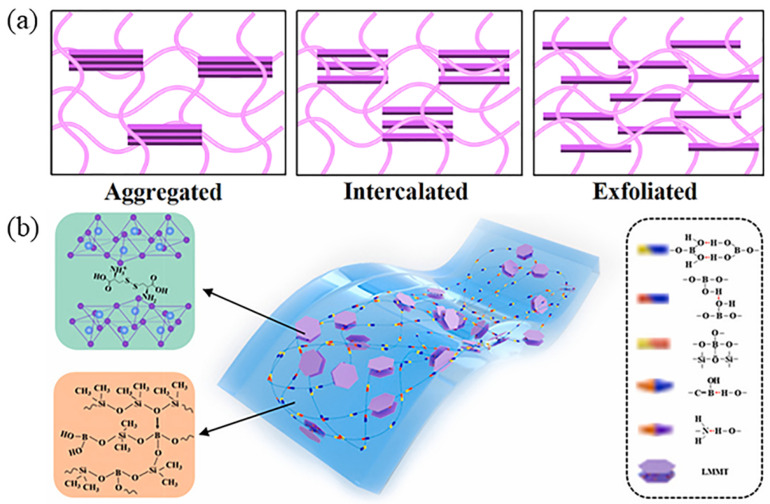
(**a**) Schematic diagram of three morphologies of polymer/clay nanocomposites; (**b**) schematic demonstration of the design of PBS/LMMT the intercoupling hydrogen bonding composite [46].

In order to achieve good dispersion of exfoliated and oriented nanosheets in polymer matrix to obtain desirable properties, lamellar fillers are usually treated with physical and chemical approaches to exfoliation as well as to improve their compatibility with polymers. For example, in a study on polystyrene (PS)/MMT nanocomposites, Huang et al. reported that by a simple mechanical stretching process, the MMT nanosheets filled in the PS matrix acquired a higher degree of orientation, and the degree of exfoliation also improved, thus effectively improving the barrier properties and other physical properties including stiffness. The addition of 2.7 vol% of MMT to the PS matrix decreased the permeability by 30% (O_2_) and 50% (H_2_O), respectively [45]. Modifications with surfactants (such as quaternary ammonium salts) also help to improve the interfacial interaction between the polymer matrix and the clay, thus contributing to the excellent mechanical and barrier properties. Zhao et al. presented a simple approach to fabricating nanocomposites by constructing coupled clusters of short hydrogen bonds between polyborosiloxane (PBS) and L-cystine-modified MMT nanosheets, resulting in weak entanglement and interfacial interactions, see Figure 3. Remarkable barrier properties (WVP ~4.74 × 10^−11^ g·m/(m^2^·s·Pa) was achieved even after repeated damage-healing cycles [46].

#### 2.4.3. Surface Barrier Coating Methods

Surface barrier coating is generally carried out by coating a high barrier inorganic (SiO*_x_*, AlO*_x_*, TiO*_x_*), organic (PVDC, PVA, and PAA), or organic/inorganic (PVA/MMT) hybrid thin layer in the surface of a polymer film by chemical vapor deposition (CVD), physical vapor deposition (PVD), dip-coating or layer-by-layer self-assembly technology [47].

##### Surface Inorganic Coating

**Figure 4:**
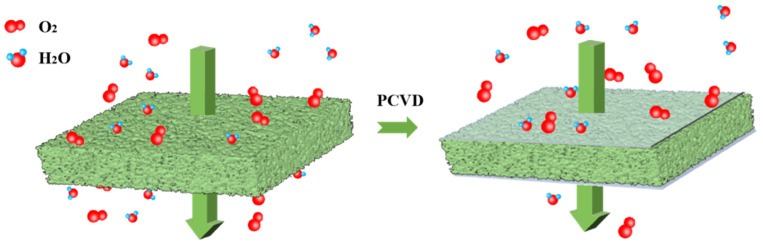
Model for the tortuous path of gas through SiO*_x_*_-_coated polymer layer.

Physical vapor deposition (PVD) and plasma-assisted chemical vapor deposition (PCVD) are commonly used to coat an inorganic layer with a thickness in the nanometer range on PET, BOPP, and other polymer substrates. For the PCVD method, metal and its oxides are melted and vaporized under high vacuum conditions so that they are cooled and deposited on the film to form a precipitated coating [48]. For example, aluminum is commonly used as a raw material for evaporation, and aluminum-coated plastic films have been widely used as barrier package materials. SiO*_x_* is another commonly used barrier coating material; a dense and uniform transparent SiO*_x_* high barrier layer can be deposited by CVD of SiO*_x_* or PCVD of gaseous organosilane and oxygen on PET, PP, or PLA, which can significantly improve the barrier performance of the films [49,50,51,52], see Figure 4.

Recently, thick inorganic films on PET (polyethylene terephthalate) and PI (polyimide) substrates have been formed at low temperatures in a method involving the application of a polysilazane coating, resulting in organic films with improved gas barrier capabilities [53,54]. Perhydropolysilazane (PHPS), which has the fundamental chemical formula (-Si(H_2_)-NH-), is the most common solution precursor. It typically undergoes pyrolysis, photolysis, hydrolysis, and oxidation reactions of the active Si-N link, N-H bond, and Si-H bond to produce silica [55]. Several authors [56,57] have studied the conversion of PHPS by UV irradiation and obtained a significant penetration reduction with thin silica layers on polymers, demonstrating the potential for PHPS-derived silica as a high-barrier coating application. Morlier et al. [53] described that a single-layer 250 nm PHPS-derived SiO*_x_* on a 50 μm PET substrate showed an OTR of 0.06 cm^3^/(m^2^·day·bar) and a WVTR of 0.2 g/m^2^·day, multilayer-stack with two SiO*_x_*-layers and polyvinyl alcohol (PVA) as an intercalated layer showed an OTR of 10^−3^ cm^3^/(m^2^·day·bar) and a WVTR of 0.02 g/(m^2^·day). Prager et al. also reported an improvement in the oxygen barrier by a factor of two orders of magnitude with a single layer of PHPS-derived SiO_x_ on a PET substrate [58].

##### Surface Organic/Inorganic Hybrid Nanocoating

Organic/inorganic hybrid nanocoatings have a “nanobrick wall” microstructure made of nanoplatelet bricks and polymeric mortar that offers porous polymer substrates a high gas barrier. Layer-by-layer (LbL) assembly, which involves immersing the substrate alternately in a solution of polyelectrolytes and a dispersion liquid of oppositely charged nanoflakes, has emerged as a leading technology for the production of these organic/inorganic hybrid nanocoatings [56]. The polyelectrolytes include polyacrylic acid (PAA) [57], anionic poly(vinylphosphonic acid) (PVPA) [59], and poly(sodium styrene-4-sulfonate) (PSS) [60] et al. The nanoflakes include sodium montmorillonite (MMT) [61], laponite (LATP) [57], vermiculite (VMT) [62], and graphene oxide (GO) [63]. It is well known that the use of nanoplatelets to impart barrier properties to those highly permeable polymers can only be maximized when the nanoflakes are oriented perpendicular to the diffusion direction of the gas molecule. The layer-by-layer assembly has the unique advantage of controlling the deposition of nanoflakes from aqueous suspensions [64], negatively or positively charged in water, nanoplatelets are easily deposited flat on a substrate due to the attraction of an oppositely charged surface [57].

Despite the advantages of the LbL self-assembly method, large-scale continuous production of large-sized samples remains a huge challenge. Recently, another approach was proposed to fabricate the organic/inorganic hybrid nanocoatings using a very simple one-step dip-coating followed by a flow-induced orientation process. The substrate membrane is immersed in a low-viscosity water-based dispersion containing inorganic nanoplatelets and polymer binder, then suspended vertically hung in an oven for drying to allow flow-induced orientation, which helps align the nanoplatelets along the flow direction on the substrate surface. During the flow-induced orientation, the nanoplatelets and the polymer chains can co-assemble to form a highly ordered layered structure with dozens of layers with only one step, and the composition of the final nanocoating can be easily adjusted, leading to a high composition flexibility and barrier property tunability [65]. This approach is easier than the LbL assembly and is suitable for continuous mass production. For example, excellent transparent PVA/LP nanocoatings have been manufactured by Sun’s group though a facile flow-induced coassembly process. Such a thin layer of PVA/LP nanocoating on both sides of the PET substrate significantly improves oxygen barrier properties while keeping the high transparency [66].

##### Surface Organic Coating

Polyvinyl alcohol (PVA) and polyacrylic acid (PAA) have excellent oxygen barrier properties, and polyvinylidene chloride (PVDC) has good both oxygen and water vapor barrier properties, but they all have poor thermal processibility; therefore, they are usually used to coat on the surface of polymer matrix films to increase barrier properties. Biaxially oriented polypropylene (BOPP) is a polymer usually used in the production of food packaging materials. Its low price and excellent thermal stability make it an attractive packaging material. Daniloski et al. compared the barrier properties of biaxially oriented coextruded polypropylene (BOPPcoex) with acrylic/polyvinylidene chloride coated biaxially oriented polypropylene ((BOPPAcPVDC) [67], and found that BOPPAcPVDC film provided better protection for fresh pork than BOPPcoex film due to its greater barrier behavior, which showed smaller permeance data (q) (0.98 to 324 cm^3^/(m^2^·day·bar)) compared with BOPPcoex (227 to 6200 cm^3^/(m^2^·day·bar)). The shelf life of fresh pork packed in BOPPAcPVDC film was doubled.

Polyethylene terephthalate (PET) has been widely used in the fields of food and pharmaceutical packaging. In order to endow the PET composite film with high barrier properties, a thin coating layer of 4,4′-diphenylmethane diisocyanate (MDI), polyacrylic acid (PAA), and borax was separately grown on the PET matrix, resulting in a dramatically decreasing of OTR value to the detection limit of commercial instruments (<0.005 cc/(m^2^/day)) [68].

#### 2.4.4. Polymer Structure/Architecture Tailoring

Polymer chemists have devised a range of strategies to generate polymers with diverse features to design their barrier qualities based on a knowledge of the chemical structure of polymers and their impact on those properties. The oxygen barrier of PVOH, for instance, can be hundreds of times greater than that of PE by substituting a “OH” for the “H” in a (-CH_2_-CH_2_-) skeleton [69]; The PT6HP-coPBL (poly (trans-hexahydrophthalide)-co-poly(butyrolactone)) copolymers’ characteristics may be tailored so that they have barrier and mechanical qualities similar to those of commonplace polymers such as PET [70].

#### 2.4.5. Crystallization and Orientation

For semicrystalline polymers with both crystalline and amorphous phases, the crystalline phase can act as an impermeable barrier to small gas molecules, such as inorganic nanofillers, so that good barrier properties can be obtained by tuning their supramolecular microstructure [71]. The orientational structure and the formation of perfect crystals are beneficial for improving the barrier properties [72]. Wang et al. [73] have demonstrated the importance of lamellar crystal arrangement in improving the gas barrier properties of polyethylene oxide (PEO) and poly(ethylene-co-acrylic acid) (EAA) alternate multilayers. They found that the oxygen permeability is reduced by two orders of magnitude when the PEO crystal forms a single, high-aspect-ratio lamella parallel to the nanolayer, compared with a multilayer with spherical PEO crystal. This encouraging result suggests that the gas barrier properties of semi-crystalline polymers can be significantly improved by tuning their lamellar arrangements.

## 3. Biodegradable Polymers and Their Potential as High Gas Barrier Packaging Materials

Over the past decades, the superior material properties of various polymers have expanded many new opportunities in lightweight packaging, agricultural fields for improved crop production, cosmetics, detergents, and more advanced applications, among many others.

**Figure 5:**
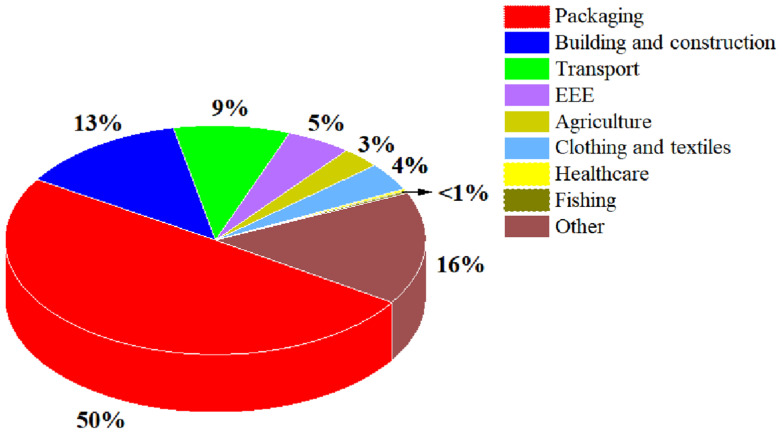
Europe plastic waste generation by all sectors in 2023 [75].

Despite the practicality and high price of plastics, environmental worries regarding plastic pollution have drawn attention on a worldwide scale. On the one hand, the manufacture and processing of plastics are energy-intensive operations that result in higher greenhouse gas emissions. On the other hand, burning plastics releases noxious emissions such as carbon monoxide, chlorine, hydrochloric acid, dioxin, furans, amines, nitrides, styrene, benzene, 1,3-butadiene, and acetaldehyde, which pose a threat to the environment as well as to public health [74]. There has been a growing awareness of the pollution caused by plastic disposal and related material solutions since the early 1970s. In 2023, according to all sector figures, more than 26 million tons of plastic waste were generated from Europe, of which around 50% originated from the packaging sector [75], as shown in Figure 5. Biodegradable plastics are a great replacement for the aforementioned non-biodegradable packaging since they can be broken down or composted after being used. Additionally, it can operate as a selective functional membrane or barrier to gases, moisture, and aromas. Due to the lack of high barrier properties comparable with traditional non-biodegradable materials, biodegradable materials are not widely used in today’s society. A comprehensive understanding of the oxygen/water vapor barrier of different biodegradable polymers and their barrier-structure co-relationships is of significant importance and can promote academic and industrial design of high-barrier sustainable packaging for future market requirements.

### 3.1. Classification of Biodegradable Polymers and Their Properties

**Figure 6:**
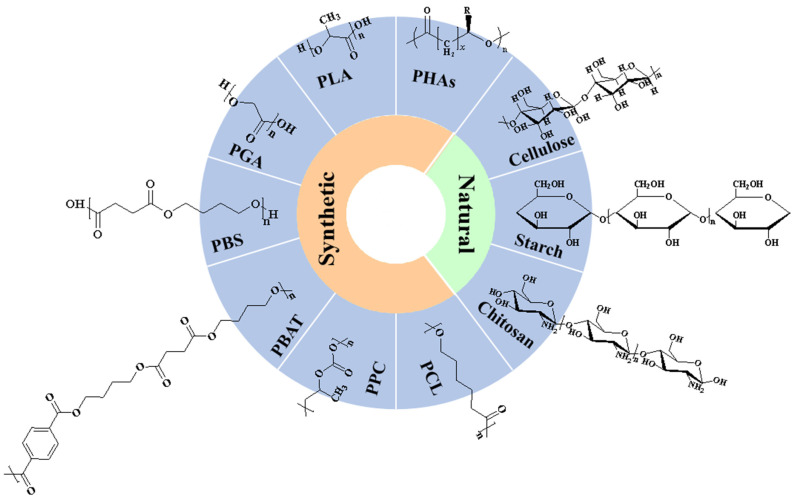
Classification and chemical structures of some represented biodegradable polymers.

According to ASTM standards D-5488-94d, biodegradability is defined as the ability to undergo decomposition into carbon dioxide, methane, water, inorganic compounds, and biomass. Biodegradable polymers can be classified as synthetic (PLA (polylactic acid), polyhydroxyalkanoates (PHAs), poly(propylene carbonate) (PPC), poly(butylene succinate) (PBS), poly (butylene adipate terephthalate) (PBAT), polycaprolactone (PCL) and natural biodegradable polymers (cellulose, starch, protein, chitosan and lipid). The chemical structure of the represented biodegradable polymers is presented in Figure 6.

Packaging materials should have good barrier, mechanical, and thermal properties to ensure that food is not damaged during storage and transportation. The barrier, mechanical, and thermal properties include water vapor transmission rate (WVTR), oxygen transmission rate (OTR), tensile strength, elongation at break, glass transition (*T_g_*) on temperature, and melting point (*T_m_*) of the materials. The shelf life of any product in a package depends primarily on the barrier properties of the packaging material. The water vapor transmission rate and oxygen transmission rate of packaging materials are critical parameters to understand in terms of the shelf-life stability of food products as water vapor and oxygen penetration across packaging boundaries affect microbial growth, product texture and functionality, overall chemical stability and pack fogging (if the vapor condenses) [76]. Mechanical properties of biodegradable polymer material also play an important role during the selection of packaging material and, consequently, in the process utilized to prepare the final product. In addition, a lot of packaging containers are commercially used at below room temperature, so it is important to assess the mechanical properties in these conditions [77]. Thermal properties of the film also play an important role during the selection of packaging material because the thermal properties determine the processing, packaging, and storage temperatures, as the polymers that are used for storage of foods at lower temperatures should have lower glass transition temperatures and those for which food materials are packed at high temperature should have a high melting point [78]. The barrier, mechanical, and thermal properties of different biodegradable polymers are listed in Table 3.

Because of either their general thermal, mechanical, and processing properties or because their oxygen or water vapor barrier properties are still not good enough to be used as high barrier packaging polymers, biodegradable polymers, despite having significant advantages in the resource and environmental aspects, have not been widely used in the field of barrier packaging. Therefore, much effort has been made in recent years to increase the overall properties of biodegradables for barrier packaging applications.

### 3.2. Research on Improving the Barrier Properties of the Biodegradable Polymers

#### 3.2.1. Cellulose-Based Biodegradable Polymers

The most common biopolymer utilized in load-bearing applications is cellulose, which may be found in plant fibers, wood products, and chemically derived films and fibers [106]. Cellulose has a respectable ability to form films, making it suitable for use in the packaging industry as large-scale films [107], nanopaper [108], or nanocomposite [109]. The high modulus, high tensile strength, and low oxygen permeability of cellulose films are its main benefits. However, cellulose films’ inadequate water vapor barrier characteristics are a result of the abundance of hydroxyl groups on the surface of the material [110]. Chemical modification, the addition of inorganic flake nano-filler, treating the surfaces of cellulose substrates with a synthetic polymer such as PVDC or a biopolymer (e.g., whey proteins, polycaprolactone, poly(lactic acid), beeswax) [111,112], or inorganic impermeable particles such as mica [113], graphene oxide [114], or montmorrilonite [115] through layer-by-layer lamination are common methods to increase the moisture barrier capacity of cellulose.

##### Chemical Modification

Chemical modification is the process of modifying the physical and chemical characteristics of cellulose particles or fibers using one or more chemical agents. There are two secondary (C2 and C3) and one primary alcohol group in each of the cellobiose moieties. These groups can undergo a variety of chemical processes, such as oxidation and substitution.

Although cellulose nanofibrils (CNF) can provide ideal barrier layers, their usage is constrained by weak rheological capabilities, brittleness, and moisture sensitivity. Kwon et al. [116] created all-cellulose nanocomposite films using cellulose nanocrystals (CNCs) and 2,2,6,6-tetramethylpiperidine-1-oxy-oxidized cellulose nanofibers (TEMPO-CNFs) via a straightforward vacuum filtration technique in order to address these difficulties. A transparent, free-standing substrate was built using TEMPO-CNFs, and the CNCs were employed as a coating material to enhance the mechanical and water vapor barrier capabilities of the finished product. The final CNC/TEMPO-CNF/CNC films demonstrated an exceptional tensile strength of 114 MPa and a comparatively low water vapor transmission rate (SWVTR) of 19 g·mm/(m^2^·day). The CNC/TEMPO-CNF/CNC films were also resistant to acetone, ethanol, tetrahydrofuran (THF), water, and other solvents. Balasubramaniam et al. [117] examined the modification of precast CNF films with saturated fatty acids lauric, palmitic, and stearic acids utilizing various modification procedures in order to increase the hydrophobicity of CNF films and preserve outstanding mechanical qualities. Surface modification and bulk modification were the two alteration methods used. The membrane created by bulk modification of CNF greatly enhances its water vapor barrier characteristics. Both modification approaches produced films with improved surface hydrophobicity. However, surface modification methods produced changed films with greater mechanical property preservation.

##### Addition of Inorganic Flake Nano-Filler

Over the past few decades, a different method for creating high gas barrier polymer films has been developed: incorporating nanoplatelets into polymers [118,119]. As multiple physical barriers to molecule diffusion, nanoplatelets significantly improve the barrier properties of polymer-based nanocomposites. Tayeb et al. proposed a novel nanocomposite film consisting of cellulose nanofibrils (CNFs)/colloidal montmorillonite nanoclay (MMT) with two cross-linking agents, namely, polyamidoamine epichlorohydrin (PAE) and acrodur thermoset acrylic resin (ACR). The combination of clay nanoplatelets and crosslinkers contributes to a denser membrane structure and restricted water passage. As a result, the water vapor transmission rates of the hybrid film were significantly reduced to 160 g/(m^2^·24·h) [120]. The morphology of the nanosheets (such as their exfoliation, dispersion, and orientation) in the polymer matrix, their intrinsic characteristics (such as high surface area and high aspect ratio), and the interfacial adhesion between the nanosheets and the polymer matrix all have a significant role in the enhancement of efficiency. Nanosheets, in particular, can increase the tortuosity of the diffusion molecules’ penetration path when placed at a height perpendicular to the diffusion direction. Layer-by-layer coating and hot pressing were used by Ren et al. to create graphene oxide nanosheet/cellulose nanofiber (GONS/CNF) nanocomposite films, and the exfoliated GONS and CNF were strongly orientated along the film direction under a powerful external shear flow field. Due to their unusual structure, GONS/CNF nanocomposites function very well as water vapor barriers and have a high oxygen content. With only 3.66 vol% GONS, the oxygen permeability coefficient of CNF film decreased by about 4 × 10^4^ times, from 5.5 × 10^−13^ to 1.4 × 10^−17^ cm^3^·cm/(cm^2^·s·Pa), and the permeability coefficient of water vapor (P_H2O_) decreased from 1.61 × 10^−12^ to 1.10 × 10^−12^ g·cm/(cm^2^·s·Pa) [121].

##### Surface Coating

The enhancement of the barrier qualities of cellulose-based films in high-humidity conditions has been the subject of certain research projects. One tactic is the creation of multilayer systems containing hydrophobic polymers, such as PP and PET, as exterior layers that can shield the cellulose layers [122]. Kim et al. [123] introduced a transparent, water-stable, high-oxygen barrier packaging film made from a combination of succinylated cellulose nanofibers (SCNF) and a fluoropolymer (FP) coating. Introducing the FP topcoat on SCNF enabled a synergistic enhancement of both oxygen barrier performance (0.1–0.3 cc/(m^2^·day·atm) at 0% RH) and stability against water-swelling. Wang et al. [124] prepared PP/CNMs/PP multilayer packaging films. CNMs maintain their high oxygen resistance at 80% RH after being laminated with PP; the water vapor transmission rate of CNC film dropped from 516 to 1.0 g/(m^2^·day). The oxygen transmission rate of CNC film at 80% RH decreased from 126 to 6.1 cm^3^/(m^2^·day). A perhydropolysilazane-derived-SiO_x_ coating layer was applied to the cellulose films by a facile dip-coating followed by a UV curing method, resulting in a significant increase in their oxygen and water vapor barrier properties. The cellulose film (35 µm in thickness) with 450 nm thick SiO*_x_* coating exhibits a low oxygen transmission rate (OTR) value of 0.82 cm^3^/(m^2^·day) and water vapor transmission rate (WVTR) value of 1.28 g/(m^2^·day) [79].

##### Layer-by-Layer Assembly

Layer-by-layer (LBL) assembly was first suggested by Decher and colleagues to build ultrathin films by alternating the deposition of components having complimentary chemical interactions [125]. The air diffusion channel was greatly extended upon the deposition of nanoplatelets in a highly orientated orientation during LBL assembly, which has been shown to be an incredibly successful strategy for enhancing the barrier qualities of the substrate films. Using quadlayers of carrageenan (CR), chitosan (CS), montmorillonite (MMT), and CS, Li et al. effectively created superhydrophobic LBL polymer-nanoclay hybrid multilayers. A paper sample modified with a wax-treated (CR/CS/MMT/CS)_2_ multilayer had a water contact angle of 151.4°. This sample of superhydrophobic paper has comparable tensile strength and strong barriers to air and water vapor compared with the original paper [126]. Zhou et al. prepared a novel nanocomposite membrane consisting of cellulose acetate (CA)/polyethyleneimine (PEI)/reduced graphene oxide (rGO)-NiCoFeO_x_) by using “molecular glue” and “nano-patching” strategies, which has excellent gas barrier properties [127]. All these studies indicated that LBL assembly is a suitable technique to improve some application properties of cellulose paper.

#### 3.2.2. Starch Based Biodegradable Polymers

Starches are inexpensive and widely accessible polysaccharides. It is made up of the branching, amorphous polymers amylopectin (poly-1,4-d-glucopyranoside and -1,6-d-glucopyranoside) and amylose (poly-1,4-d-glucopyranoside), which is a linear crystalline polymer. Depending on the source, the levels of amylose and amylopectin in starch can range from 10 to 20 percent and 80 to 90 percent, respectively. Biodegradable polymers are made using a variety of starches, including potato, cassava, rice, corn, and tapioca. However, starch has numerous significant flaws that prevent the creation of goods based on it, such as poor mechanical behavior and moisture resistance [128]. Moreover, plasticized starch suffers from recrystallization and retrograde phenomena that affect the stability of the mechanical properties over time [129]. Therefore, several strategies have been proposed to improve the properties of starch-based biodegradable plastics, such as chemical modification, cross-linking, blending with various biopolymers and certain additives, the use of different plasticizers, and the development of nanocomposites [130].

In a recent study, Dai et al. [131] assessed the effects of various plant-derived starches (waxy corn, cassava, sweet potato, potato, wheat, and corn) and various modified cassava starches (esterified cassava starch, cross-linked cassava starch, and oxidized cassava starch) on the physical and chemical characteristics of starch-based films used in food packaging. Adipic acid cross-linked cassava starch films were found to have better water vapor barrier qualities than other modified starch films and unmodified starch films. Cross-linked cassava starch film has a much lower water vapor permeability than other native starch films and oxidized cassava starch film.

Other biodegradable polymers, such as polyvinyl alcohol and chitosan, are mixed with the starch to improve its mechanical and gas barrier properties. Glycerol-plasticized acetylated corn starch films were developed by Jiménez-Regalado et al. [132] using a casting method, and the impact of incorporating chitosan (TPS:CH) in various proportions was studied. Chitosan-protonated amino groups promoted the formation of intermolecular bonds and improved the gas barrier properties of starch films.

The kind and amount of the plasticizer are also important determinants of how starch-based materials behave as barriers. González et al. [133] created thermoplastic starch (TPS) films using a typical maize starch matrix utilizing the extrusion/compression process and plasticizers glycerol, 1,3-propanediolane, and D-isosorbide. They discovered that the WVP did not differ significantly when different plasticizers were used but that the OP was significantly influenced by the plasticizer, and the lowest OP value of 6 ± 3 cm^3^·μm/(m^2^·day·kPa) was obtained for TPS-I (sample plasticized with D-isosorbide), which was twenty times lower compared with that obtained for TPS-G (sample plasticized glycerol). In addition, TPS nanocomposite films were prepared incorporating waxy starch nanocrystals (WSNC) and cellulose nanocrystals (CNC) into TPS-G; the WSNC showed greater effectiveness in decreasing the permeability against O_2_ molecules due to their platelet-shape morphology. The OP value reduced from 108 ± 35 cm^3^·μm/(m^2^·day·kPa) for TPS-G to 20 ± 3 (TPS-G5) cm^3^·μm/(m^2^·day·kPa) for the TPS nanocomposite with 5 wt% WSNC.

To tailor the properties of thermoplastic starch, with a particular focus on improving their barrier properties without compromising their transparency and biodegradability, Fabra et al. [134] produced thermoplastic corn starch (TPCS) nanobiocomposites containing bacterial cellulose nanowhiskers (BCNW) by direct melt-mixing. The addition of BCNW to TPCS-based films resulted in a great improvement in the mechanical properties as well as barrier properties due to the strong nanofiller-matrix adhesion by hydrogen bonding. Subsequently, the TPCS nanobiocomposites were successfully hydrophobized by coating them with electrospun poly(3-hydroxybutyrate) (PHB) and homogenizing by annealing, obtaining multilayer structures with further improved water vapor barrier properties and helping keep good oxygen barrier properties of TPCS at high humidity. Ruamcharoen et al. [135] incorporated 10% natural rubber (NR) to toughen cassava starch (CS), and nanoclays were introduced into the composites as the reinforcing or compatibilizing agent as well as barrier-enhancing agent. Three kinds of nanoclays, that is, montmorillonite (MMT), kaolinite (KAO), and intercalated kaolinite (DKAO), were used at contents of 2, 4, 6, and 8 wt%, respectively. It was found that the introduction of clays significantly improved both the mechanical and water vapor barrier properties of the pristine starch and CS/NR film. The best improvement was achieved for 4 wt% of MMT addition due to well-dispersed MMT nanosheets, the strong interaction of MMT with starch, and the formation of a tortuous path. Reductions of 53 and 68% in the WVT of the CS and CS/NR films with the loading of 4 wt% of MMT were obtained.

#### 3.2.3. Protein Based Biodegradable Polymers

Protein film-forming components are isolated from a variety of animal and plant sources, such as animal muscles and tissues, oilseeds, milk, soybeans, wheat, corn, and grains, where each contains a different composition, structure, and functionality. For this reason, protein-based films have been studied extensively. The mechanical, barrier, and thermal properties of protein-based packaging and their capabilities in preserving foodstuffs have been comprehensively investigated over the past decade [136]. In the food packaging industry, films made from protein polymers are used as edible films so that they can be consumed along with the food. In non-food packaging, polymers of keratin, casein, zein, gelatin, and soy–protein, etc., may play a crucial role in the development of various commercial products such as shopping bags, mulch films, and flushable hygiene products, etc. [137,138].

However, the barrier properties of proteins against gases such as oxygen, carbon dioxide, and water vapor are not effective enough to be competitive with the respective petroleum-based barrier plastics. This can be attributed, in particular, to their sensitivity to moisture. In addition, films made from whey protein showed relatively poor mechanical properties (tensile strength 4.38 MPa [85]) compared with synthetic films.

The mechanical properties of protein polymer can be further enhanced by cross-linking with “cross-linking agents”, those cross-linking agents including glutaraldehyde, glyoxal, phenolic components such as gallic acid, tannic acid, ferulic acid, and enzymes, etc. [139], or blending them with chitosan [140], starch [141], and pectin [142]. Parsaei et al. [143] evaluated the effects of caffeic acid (CA) and tannic acid (TA) as cross-linking agents on the mechanical and physicochemical properties of cold-water fish gelatin films. The incorporation of phenolic compounds into the gelatin films resulted in a hydrogen bond between the gelatine polypeptide chain and the phenolic compound. In this way, the structure of the gelatine film becomes compacter, and WVP and OP are reduced by 32% and 44%, respectively, for the 5% TA cross-linked film as compared with uncross-linked film. Meanwhile, the incorporation of TA and CA into gelatin films also increased mechanical strength from ~28 to ~50 MPa. Lee et al. [144] prepared active bio-active gelatin/chitosan nanoparticles (CSNPs) composite films with chicken skin gelatin; due to the intermolecular interactions between the two biopolymers, the mechanical and barrier properties of protein-based membranes can be improved, WVP value for the film with CSNPs concentration of 6% was significantly decreased (from 2.72 to 1.31 g·mm/(h·m^2^·kPa)) compared with films without CSNPs; meanwhile, the tensile strength increased from 2.29 to 4.22 MPa.

Incorporation of nanofillers or nano-reinforcements into protein films is also an effective strategy to improve their water vapor barrier and mechanical properties. The improvement effect of nanofillers on the physical properties of films is attributed to the high surface area/volume ratio of the nanofillers and the creation of strong interfacial interactions between the polymer matrix and the nanofillers. Amjadi et al. [145] investigated the effects of the dimensions and morphology of nanofillers (sodium montmorillonite (MMT), cellulose nanofibers (CNF), and titanium dioxide nanoparticles (TiO_2_NPs)) on the mechanical properties and release profile of the cinnamon essential oil (CEO) activated gelatin-based films. The WVP of the films exhibited a significant decrease upon incorporation of nanofillers. In particular, MMT-incorporated active film exhibited the lowest WVP (1.5 × 10^−10^ g/(m·s·Pa)).

In addition, by combining proteins with other biopolymers that contain eligible barrier properties such as polysaccharides, lipids, and/or other proteins, it is possible to capitalize on the specific functional characteristics of each component [146]. For instance, with the addition of hydrophobic lipids such as oils [147] and waxes [148] to the formulation of protein-derived films, the moisture permeability of the films declined significantly.

#### 3.2.4. Poly(Lactic Acid)-Based Biodegradable Polymers

Poly(lactic acid) (PLA) is a synthetic biodegradable polymer derived from renewable resources such as sugar beet, corn, sugar cane, potato, cassava, wheat straw, bagasse, or wood chips that has gained intensive attention in recent years [149]. Although high molecular weight, good processability, and biodegradability make PLA a potential green packaging material [150], it still has some limitations for food packaging usage, such as poor thermal and mechanical properties, low crystallization rate, poor melt strength, moderate water vapor barrier properties, and poor oxygen barrier properties. Various studies have reported on blending PLA with immiscible biopolymers such as nanocellulose fibrils, polyhydroxybutyrate (PHB), and polybutylene succinate (PBS) to improve its crystallization and mechanical properties [151,152]. Jung et al. [153] improved the tensile strength and flexibility as well as barrier properties of the PLA by the incorporation of cellulose nanofibers (CNF) into the PLA matrix. CNF was first homogenized in a triethyl citrate/ethanol mixture and then blended with PLA to allow uniform dispersal. Specifically, the oxygen permeability was increased up to 47.3% (16.99 cm^3^·mm/(m^2^·day·atm)) with a loading of 1 wt% of CNF in the PLA matrix.

Other attempts have been made to improve the properties of PLA significantly through the introduction of different kinds of inorganic nanoclays, carbon-based nanomaterials, metallic nanoparticles, etc. [154]. Recently, Yang et al. [155] proposed a method for enhancing the barrier properties of PLA by incorporating a 3-aminopropyltriethoxysilane (APTES)-modified MgAl-layered double hydroxide (LDH) nanosheet into the PLA matrix through scraping the coating solution composed of APTES@LDH and PLA. The OTR of APTES@LDH (5%)/PLA and APTES@LDH(10%)/PLA hybrid films with thickness of around 60 μm are lower than the testing limit of the instrument (<0.005 cm^3^/(m^2^·day·atm)) and the WVTR of the APTES@LDH (10%)/PLA hybrid film is only 0.026 g/(m^2^·day·atm), 94.1% lower than that of the pure PLA film.

Recently, Prof. Sun’s research group developed a facile flow-induced coassembly technique to fabricate organic/inorganic hybrid nanocoatings containing a high concentration of well-aligned nanosheet, which mimics the structure of nacre [13]. They prepared a poly(vinyl alcohol) (PVA)/α-zirconium phosphate (ZrP) aqueous dispersion where the ZrP was exfoliated into single-layer nanosheets. Then, the PVA/ZrP was dip-coated on PLA films, and the coated substrate films were vertically hanged, followed by chemical crosslinking with glutaraldehyde (GA), during which a high-level orientation of the ZrP nanosheets was induced. As a result, the OTR and WVTR of the coated PLA film (20 μm in thickness) decreased from 845.6 cm^3^/(m^2^·day·atm) and 107.0 g/(m^2^·day) of the uncoated PLA film to 2.0 cm^3^/(m^2^·day·atm) and 22.5 g/(m^2^·day), respectively [91].

As PLA is a semicrystalline polymer, barrier properties could also be adjusted by tailoring their super molecular microstructure of the crystalline and amorphous phases. Bai et al. [156] reported a novel and simple strategy to greatly increase the oxygen barrier property of PLA by constructing parallel aligned shish-kebab-like crystals with the aid of a fibrillar nucleating agent. They demonstrate that the fibrillar nucleating agent can change the crystallization behavior of PLA from isotopic spherulitic crystals to unique shish-kebab-like crystals, and the shear flow during the compression molding can induce the parallel alignment of the shish-kebab-like crystals along the surface direction. More importantly, at the later stage of the crystallization, the growing lamellae are found to interpenetrate and tightly interlock with each other at the boundary regions of the shish-kebab-like crystals, forming a densely packed nanobrick wall structure and thus endow the PLA with an unprecedentedly low oxygen permeability. In order to explore the influence of the structure of the amorphous phase on the barrier properties of PLA, Marta Safandowska et al. [92] modified PLA with low molecular weight compounds, such as glycerol (Gly), triethyl citrate (TEC), and polyethylene glycol (PEG) by melt blending. It was found that the incorporation of small amounts of modifier (0.5–1.5 wt%) in the PLA matrix did not affect the degree of crystallinity but densified the packing of polymer chains, resulting in an improved oxygen barrier performance.

#### 3.2.5. Polyhydroxyalkanoates (PHAs)-Based Biodegradable Polymers

Nowadays, the applications of PHAs in biodegradable packaging fields include containers, bottles, sheets, films, fibers, and coatings. Polyhydroxybutyrate (PHB) is one of the popular PHAs; however, the application of PHB has been limited due to its high production cost, brittle nature, and low thermal stability. To improve these physical adversities, copolymers known as PHBV or PHBH were obtained by insertion of 3-hydroxyvalerate or 3-hydroxyhexanoate units into PHB main chain, which are widely used due to their greater flexibility and wider processing temperature window [157,158]. Moreover, for PHBV copolymers, an extremely good balance of barrier properties towards oxygen and water vapor molecules is reported [159].

The blending of PHAs with other biopolymers is another effort to improve the overall properties of PHAs [160,161]. For example, poly(butylene succinate-co-adipate) (PBSA) and PHB blends have been comparatively evaluated for their mechanical and gas barrier properties, and the results showed that both OP and WVP decreased as a function of PHB content [162]. Unfortunately, PHAs are generally not miscible with polysaccharides and proteins due to the poor interfacial adhesion between them. To address this aspect, multilayer films can be prepared, which have fewer compatibility problems than those experienced in the fabrication of blend films. Eslami et al. [163] prepared a novel biodegradable sandwiching assembly of two outer layers of poly(3-hydroxybutyrate-co-3-hydroxyvalerate) (PHBV) and a core layer of thermoplastic starch (TPS) or maleated TPS or their blends with PHBV (80/20). The tensile test revealed that samples with a core composed of a mixture of maleated TPS and PHBV were the strongest, with a modulus as high as 178 MPa. The WVPR of the tri-layer structures was as low as 20.2 g/(m^2^·d), and the OP was below the detection limit of a bubble flow meter.

Recently, the addition of small amounts of inorganic fillers into the PHAs’ matrixes is a promising method to increase the mechanical and thermal performances of the PHAs [164]. These inorganic fillers act not only as reinforcing agents but also as nucleating agents, providing heterogeneous nucleation of the polymer and allowing for more rapid crystallization. Many inorganic nucleating agents have been studied so far, including multi-walled carbon nanotubes (MWCNT), terbium oxide, nitride, and so on. Yan et al. [165] used montmorillonite (MMT) as the nucleating agent for poly(3-hydroxybutyrate-co-3-hydroxyhexanoate) (PHBH) and found that MMT played a heterogeneous nucleation role in the crystallization of PHBV, not only increasing the crystallization rate but also narrowing the crystallization peak width of PHBH. Compared with PHBH, the tensile strength and elastic modulus of MMT/PHBH biocomposite with 1 wt% MMT increased by 111.2% and 182.5%, respectively. In addition, the OP and WVP of the composite decreased by 43.9% and 6.9%, respectively.

#### 3.2.6. Poly(Propylene Carbonate) (PPC)-Based Biodegradable Polymers

Poly(propylene carbonate) (PPC), a biodegradable polymer synthesized by alternating copolymerization of carbon dioxide (CO_2_) and propylene oxide (PO), has attracted considerable interest in both academia and industry [166]. The polar carbonate linkages contained in the PPC backbone create a considerably closer arrangement of molecular chains and a greatly smaller free volume; thus, PPC has a relatively high density of 1.3 g/cm^3^. Pure PPC has good barrier performance, the OP (23 °C, 0% RH) and WVP (23 °C, 85% RH) of pure PPC is ~2.25 cm^3^·mm/(m^2^·24 h) and ~1.05 g·mm/(m^2^·24 h), respectively [96]. Therefore, PPC is a promising biodegradable polymer with potential applications in the packaging sector. Nevertheless, PPC is still not competitive enough in barrier properties compared with high-barrier polymers such as EVOH, PVA, and PVDC [167].

In recent years, in order to extend the application range of PPC, great efforts have been made to improve the mechanical, thermal as well as barrier properties of PPC, including chemically modifying its end group, blending it with other polymers (starch, PLA, PHBH, and PHB) or introducing inorganic fillers. A series of composite films of PPC/aluminum flake (ALF) with different ALF contents were prepared by Meng’s group [96]. The WVP and OP of the composite films decrease continuously, with ALF contents increasing up to 5 wt %, which are 75.2% and 32.4% of the pure PPC, respectively. In addition, the tensile strength and thermal properties of the PPC are also improved by the incorporation of ALF particles. Li et al. [95] prepared a series of PPC/organic modified layered double hydroxide (OLDH) composite films with different contents of OLDH via melt blending. The tensile strength and gas barrier properties of PPC are obviously enhanced with the incorporation of OLDH. Compared with pure PPC, the OP and WVP were reduced by 54% and 17%, respectively, while tensile strength increased by 83% with 2% OLDH addition.

#### 3.2.7. Polybutylene Adipate Terephthalate (PBAT)-Based Biodegradable Polymers

Poly(butylene adipate-co-terephthalate) (PBAT) is a semicrystalline copolyester obtained from the copolymerization of 1,4-butanediol, adipic acid, and dimethyl terephthalate. Although derived from oil-based resources, it can be completely degraded with the help of natural enzymes. It possesses similar thermal and mechanical properties to polyethylene (PE) and is a flexible plastic suitable for film blowing [168]. However, the inferior gas barrier properties of pure PBAT limit its application as a barrier packaging material.

Many methods have been reported for PBAT modification, including blending with other low-cost biodegradable polymers and multi-layer coextrusion. As PPC is a biodegradable polymer with good barrier properties and is cheaper than PBAT, it is extensively used to blend with PBAT to tailor the properties of the polymer blends for diverse applications. To maximize the advantages of blends, interfacial modification of PBAT/PPC blends is needed to increase the interfacial interaction between PBAT and PPC. Zhao et al. [100] studied the properties of PBAT/PPC films with different component ratios and the addition of an epoxy compound ADR4488 as a chain extender on the properties of PBAT70/PPC30 blends. A reduction in the melt viscosity and a significant improvement in the water vapor barrier performance was observed with the introduction of PPC. The addition of ADR improved the compatibility between PBAT and PPC and thus increased the thermal stability and mechanical strength of the blends without affecting the water barrier property. Xie et al. [99] also investigated the effect of triglycidyl isocyanurate (TGIC) bearing three epoxy groups as a reactive compatibilizer for PBAT/PPC (50/50) blends. The PBAT/PPC/TGIC films with 1 phr TGIC prepared via blowing process exhibited a tensile strength of 30.8 MPa and an elongation at break of 860%, which were 136% and 142% of those of PBAT/PPC, respectively. Meanwhile, the water vapor transmission rate was reduced by 33% at this content, reaching 1.32 g·mm/(m^2^·24 h). Xu et al. [169] prepared PBAT/PPC/PBAT tri-layer films using the multi-layer coextrusion method. The tri-layer films showed good interface adhesion between PPC layer and PBAT layer, and the lowest oxygen permeation of 9.5 × 10^−15^ cm^3^·cm/(cm^2^·s·Pa) was obtained at a maximal PPC layer thickness of about 12 μm.

Incorporating inorganic nanosheets into the PBAT matrix is also employed to improve its barrier properties. Debeli et al. [101] reported PBAT/montmorillonite (MMT) nanocomposite films with excellent oxygen barrier properties, which were prepared by exfoliating the MMT in a water-dispersible sulfonated PBAT matrix. At higher MMT loadings of 28–32 vol%, a network-like MMT morphology was formed, and nanocomposite film with 32 vol% MMT exhibited an OP of 7.5 × 10^−16^ cm^3^·cm/(cm^2^·s·Pa) and a WVP of 1.57 × 10^−11^ g·m/(m^2^·s·Pa). Compared with based PBAT, the oxygen barrier property of the PBAT/MMT nanocomposite was improved by two orders of magnitude.

#### 3.2.8. Polycaprolactone (PCL)-Based Biodegradable Polymers

Polycaprolactone (PCL) is a flexible biodegradable polymer, which is obtained by ring-opening polymerization through a petrochemical pathway. Apart from being biodegradable, PCL has many advantages over other biodegradable polymers, such as low viscosity, easy processing, good solvent resistance, and good mechanical performance [170]. However, it is costly and also has a major drawback: it has a high permeability to gases and water vapor, making this polymer unsuitable for high-barrier packaging applications [171].

One strategy to reduce the permeability of PCL without sacrificing biodegradability is to blend it with another biodegradable polymer with good barrier character using classical routes (solution or extrusion) [172]. PPC is undoubtedly one of the best choices. Blend films of PCL and PPC were prepared using a uniaxial-stretching extrusion process by Cheng et al. [86]. The OPs of the blended film decreased by 42.7% and 64.6% compared with neat PCL when the PPC blending ratio was 20% and 50%, respectively, and the WVP decreased from 1.83 × 10^−5^ to 1.03 × 10^−5^ (g·m/(m^2^·d·Pa)) with an increase in PPC content from 0 to 20 wt%. In addition, the shelf life of button mushrooms packaged with PCL/PPC blending film is longer than that packaged with neat PCL films.

The properties of PCL and their blends can also be modulated by the addition of various nanofillers, such as clays, graphene, carbon nanotubes, and metallic oxide nanoparticles [5]. Recently, Bujok et al. synthesized trihexyl(tetradecyl)phosphonium decanoate ionic liquid (IL) functionalized ZnO nanoparticles (IL-ZnONPs) and layered double hydroxides (IL-LDH), and then microwave-assisted in situ ring-opening polymerization of ε-caprolactone (εCL) in the presence of dispersed IL-ZnONPs or IL-LDH was used for preparation of PCL/ZnONPs and PCL/LDH nanocomposites, which leads to the homogenous dispersion of nanofiller in the PCL matrix and the formation of large PCL crystallites, resulting in improved thermal, mechanical and barrier properties of the nanocomposite [87,88].

Glycerol tristearate (C18) has good gas barrier properties due to its hydrophobic nature and closely stacked lattice arrangement; hence, it is used as an additive to increase the barrier properties of PCL. The water vapor and oxygen barrier properties of PCL/C18 composites with 30% C18 content were reported to have increased by 81.4% and 39.2%, respectively [173]. However, due to the zero-dimensional dispersion morphology, the barrier performance was enhanced only at high C18 content with the sacrifice of its mechanical properties. Recently, Ding’s team reported an effective strategy for preparing PCL/C18 composites with a two-dimensional sheet-like C18 dispersion phase by multistage biaxial-stretching extrusion. Compared with conventional PCL/C18 blends with the same content of C18 (10%), the penetration path of the small molecules was significantly increased, resulting in the improvement of water vapor and oxygen barrier properties by 39.6% and 63.7%, respectively. Furthermore, when the relative humidity increased from 50% to 90%, the WVP of the sample only increased slightly from 6.94 × 10^−14^ to 7.66 × 10^−14^ g·cm/(cm^2^·s·Pa), exhibiting excellent stability to high humidity [174].

## 4. Summary and Outlook

With the global concern of plastic pollution, biodegradable polymers from synthetic and natural are continuously being developed, and they show great potential for major uses in packaging for their sustainability and biodegradability. However, the drawbacks of biodegradable plastics, such as the brittle nature of PLA, high cost and poor barrier properties of PCL and PHAs, poor stabilization and mechanical properties of starch, insufficient thermal stability of PPC, poor mechanical strength and barrier properties of PBAT and poor processibility of cellulose, limit their applications in packaging field.

Various strategies have been reported to improve the oxygen/water vapor barrier as well as mechanical/thermal properties of biodegradable films, such as polymeric blending, chemical and physical modifications, the addition of nanosheets, and organic/inorganic coating technologies such as flow-induced co-assembly and layer-by-layer assembly. While promising successes in improving barrier properties of biodegradable plastics have been achieved with the discussed strategies, challenges remain prevalent with developing new techniques and properties in the future to meet the requirement of barrier packaging applications in terms of both performance and cost.

1. The price or cost-effectiveness of biodegradable material manufacturing needs to be addressed in advance. The prices of petroleum-based plastics such as PP, PE, and PET are around $1.1–1.4/Kg, while the average prices of commercial PHAs, PCL, PBS, PLA, PPC, and PBAT are around $6.9/Kg, $6.4/Kg, $3.3/Kg, $3.0/Kg, $3.2/Kg, and $1.7/Kg, in turn. The cost of the cheapest biodegradable plastic is still higher than the current widely used petroleum-based plastic. To address this issue, two key aspects can be considered: first is exploring new technologies towards high purity and low-cost monomers so as to develop new low-cost biodegradable plastics products; Secondly, increasing production capacity through stimulating market demand will also play a vital role in cutting down the production cost.
2. The construction of organic/inorganic hybrid nanocoatings has emerged as a facile and effective strategy to enhance the oxygen barrier property of biodegradable materials. However, due to the coating layer being mostly composed of hydrophilic inorganic nanosheets and hydrophilic polymers, the barrier against water vapor is not as expected. Therefore, more research attention should be paid to the design of all-organic or all-polymer multilayer degradable hydrophobic coatings for enhancing the water vapor barrier properties of biodegradable polymer films.
3. A more recent strategy for overcoming some drawbacks of biodegradable is the preparation of multilayer polymeric films by coextrusion or blending with different biodegradable polymers. The application of this strategy is restricted by the limited variety and compatibility of commercial biodegradable polymers. So, the development of more biodegradable polymers and the research on compatibility improving of biodegradable polymers will continue to rise in the near future.
4. Multiple strategies should be simultaneously applied to obtain a biodegradable packaging material with comprehensive performances, such as biodegradable polymer blending to tailor the mechanical and thermal properties combined with a surface coating to increase the barrier properties further.
5. Although previous research has achieved success in improving the barrier, mechanical, or thermal properties of biodegradable materials, few real application properties of these materials for packaging were reported, which is one of the targets to be explored in the future.

The market for plastic barrier packaging is huge, and there is great potential for biodegradable polymers to be used in barrier packaging to make better use of agricultural waste and address the fossil fuel shortages, solid waste management, health hazards, and environmental issues that plastics have. In all, innovation driven by biodegradable materials will contribute to a sustainable environment, economy, and society.

## Figures and Tables

**Table 1:** Representing method and common units of oxygen and water vapor barrier property.

	Representing Method	Units	Standard
Oxygen barrier performance	Oxygen Permeability, OP	cm^3^/(m·24 h·0.1 MPa)	GBT19789-2021
		cm^3^·cm/(m^2^·s·Pa)	GBT1038.1-2022
	Oxygen Transmission Rate, OTR	cm^3^/(m^2^·24 h·0.1 MPa)	GBT19789-2021
		cm^3^/(m^2^·day·Pa)	GBT1038.1-2022
Water vapor barrier performance	Water Vapor Permeability, WVP	g·cm/(m^2^·s·Pa)	GBT 1037-1988
		g·mm/(m^2^·day)	GBT 21529-2008
	Water Vapor Transmission Rate, WVTR	g/(m^2^·24 h)	GBT 1037-1988
		g/(m^2^·24 h)	GBT 21529-2008

**Table 2:** WVP, OP, density, and crystallinity (χ) of some commonly used polymer package materials.

Entry	Sample	OP (cm^3^·mm/(m^2^·day))	WVP (g·mm/(m^2^·day))	Density (g/cm^3^)	Crystallinity (χ)	Reference
1	PET	1.536 (23 °C, 0% RH)	0.098 (23 °C, 50% RH)	1.37	30~40	[13,14]
2	BOPP	37.2 (23 °C, 0% RH)	0.022 (23 °C, 50% RH)	0.91~0.92	45~60	[13,15]
3	HDPE	64.262 (23 °C, 0% RH)	0.015 (23 °C, 50% RH)	0.95~0.97	57~79	[13,16]
4	LDPE	102.87 (23 °C, 0% RH)	0.053 (23 °C, 50% RH)	0.91~0.93	27~28	[13,17]
5	PA	1.015 ± 0.006 (23 °C, 0% RH)	0.099 ± 0.004 (38 °C, 90% RH)	1.0~1.1	20~25	[18,19]
6	PVDC	0.012 (23 °C, 0% RH)	0.014 (38 °C, 100% RH)	1.96	50~80	[20]
7	EVOH	0.0023 (23 °C, 0% RH)	70.848 (23 °C, 95% RH)	1.13~1.21	34~36	[21,22,23]

**Table 3:** Water vapor and oxygen barrier, thermal and mechanical properties of biodegradable polymers.

Entry	Sample	OP	WVP	T_g_/°C ^h^	T_m_/°C ^i^	Tensile Strength (MPa)	Ultimate Strain (%)	Reference
1	Cellulose	0.365 ^a,c^	2.305 ^e,f^	--	--	110	23	[79]
2	Starch	0.06–14 ^a^	0.07–0.7 ^e^	100~101	209~211	1~15	2~30	[80,81,82,83,84]
3	Whey protein isolate	0.0053 ^b,c^	526.287 ^e,g^	--	--	4.38	75.83	[85]
4	PCL	60~80 ^a^	1.8~7.2 × 10^3 e^	(−65~−50)	55~56	24~33	200~450	[86,87,88]
5	PLA	18~25 ^a^	1~5 ^e^	55~62	148~166	20~75	2~12	[13,89,90,91,92,93]
6	mcl-PHA	197 ^b,d^	-	(−66~−16)	25~55	3.9 ± 0.69	273 ± 27	[94]
7	PPC	2~3 ^ac^	1~5 ^e^	17~32	--	3~15	500~1000	[95,96,97]
8	PBAT	49~104 ^a^	6~15 ^e^	(−31~−28)	120~121	30~40	490~1700	[98,99,100,101,102]
9	PBS	4~30 ^a^	5~15 ^e^	(−36~−33)	104~113	30~50	15~185	[103,104,105]

^a^ cm^3^·mm/(m^2^·day); ^b^ cm^3^·mm/(m^2^·day·atm), ^c^ 23 °C, 0% RH; ^d^ 23 °C, 80% RH; ^e^ g·mm/(m^2^·day); ^f^ 37.8 °C, 85% RH; ^g^ 37.8 °C, 100%RH; ^h^ Glass transition temperature; ^i^ Melting temperature.

## Data Availability

The authors do not have permission to share data.
